# Ice recrystallization is strongly inhibited when antifreeze proteins bind to multiple ice planes

**DOI:** 10.1038/s41598-018-36546-2

**Published:** 2019-02-13

**Authors:** Anika T. Rahman, Tatsuya Arai, Akari Yamauchi, Ai Miura, Hidemasa Kondo, Yasushi Ohyama, Sakae Tsuda

**Affiliations:** 1 Graduate School of Life Science, Hokkaido University, Sapporo, 060-0810 Japan; 2 Bioproduction Research Institute, National Institute of Advanced Industrial Science and Technology (AIST), Sapporo, 062-8517 Japan; 3 OPERANDO Open Innovation Laboratory, National Institute of Advanced Industrial Science and Technology (AIST), Tsukuba, 305-8563 Japan

## Abstract

Ice recrystallization is a phenomenon observed as the increase in ice crystal size within an already frozen material. Antifreeze proteins (AFPs), a class of proteins capable of arresting ice crystal growth, are known to inhibit this phenomenon even at sub milli-molar concentrations. A tremendous range in the possible applications of AFPs is hence expected in both medical and industrial fields, while a key determinant of the ice recrystallization inhibition (IRI) is hardly understood. Here, IRI efficiency and ice plane affinity were examined for the wild-type AFPI–III, a defective AFPIII isoform, and a fungal AFP isoform. To simplify the IRI analysis using the formal representation of Ostwald-ripening (*r*^3^ = *r*_0_^3^ + *kt*), we monitored specific ice grains exhibiting only uniform growth, for which maximum Feret diameter was measured. The cube of an ice grain’s radius (*r*^3^) increased proportionately with time (*t*), and its slope gave the recrystallization rate (*k*). There was a significant difference in the IRI efficiency between the samples, and the fungal AFP possessing the activity with the smallest amount (0.27 μM) exhibited an affinity to multiple ice planes. These results suggest that the IRI efficiency is maximized when AFPs bind to a whole set of ice planes.

## Introduction

A general ice block is made of an infinite number of single ice crystals, whose size and shape are not uniform^1^. Large ice crystals grow at the expense of smaller ones through the process called “Ice Recrystallization”. The recrystallization rate is evaluated using Ostwald-ripening principle, represented formally by *r*^3^ = *r*_0_^3^ + *kt*, where *r*_0_ is the radius of an averaged ice grain size at *t* = 0, and *k* is the recrystallization rate (μm^3^·min^−1^)^2^. The resultant expansion in size of ice grains physically destroys the texture of any frozen materials from both inside and outside, especially during the freeze-thaw cycle conducted at temperatures around zero^3,4^. Antifreeze protein (AFP) and its mimics are known to possess the ability of ice recrystallization inhibition (IRI)^5–7^. The mechanism of inhibition and what factors determine a strong IRI efficiency however, are not understood, although many tools and software have been developed to appreciate thousands of ice grains in photomicroscope snapshots^7–12^. Here, an alternative method, which monitors just 13–15 ice grains that exhibit only uniform growth, was examined in a video file. Consistency between the IRI efficiency parameter obtained from this method and from previous knowledge is discussed in light of the ice-binding properties of each AFP species.

**Figure 1:**
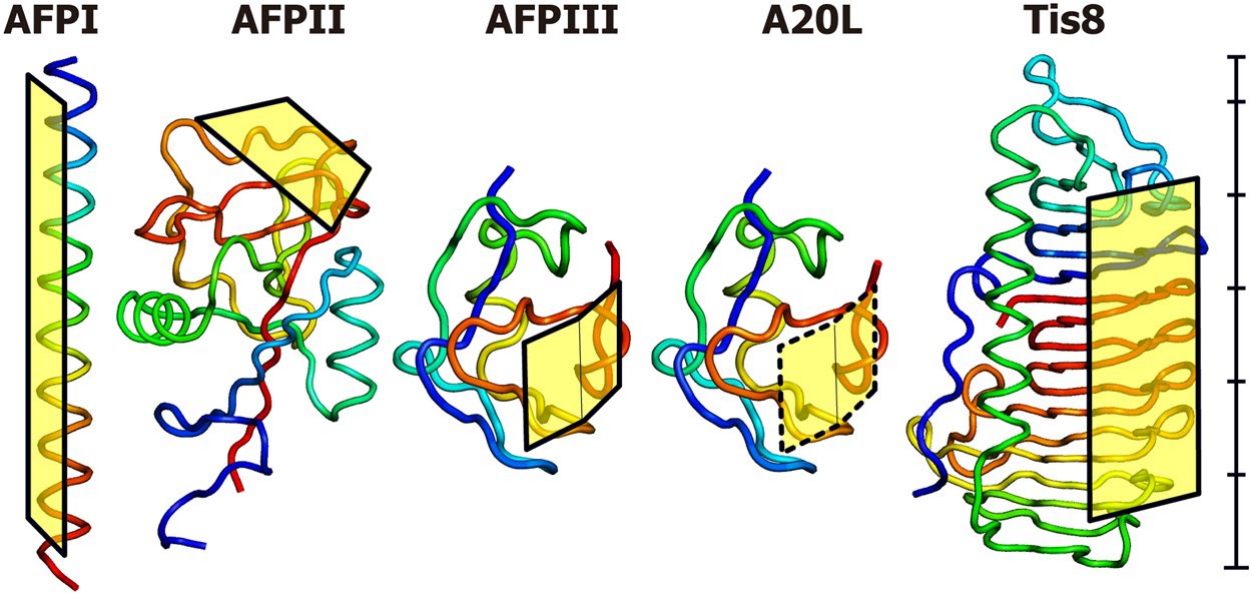
Structural models created with Pymol of the AFPs examined in this study^53,54^. A rod-like α-helical motif (1WFB.pdb) was speculated for AFPI from barfin plaice. AFPII from longsnout poacher has an elongated globular structure (2ZIB.pdb). AFPIII (5XQN.pdb) and its defective mutant A20L (5XQV.pdb) from notched-fin eelpout form identical structures except for the 20^th^ residue. Tis8, from snow-mold fungus, forms another globular structure through its β-helical backbone (5B5H.pdb). Squares indicate the ice-binding-regions, though it is not perfectly formed for A20L (hatched square). The structures were adjusted in scale, and a 55 Å scale bar is shown beside Tis8.

AFPs initially isolated from the polar sea area have also been discovered in various organisms living in mid-latitude waters, whose amino acid sequences are partially different^13,14^. Examples include AFP I from barfin plaice *Liposetta pinnifasciata*, an alanine-rich amphipathic α-helical polypeptide (Mw = 3.5 kDa), AFP II from longsnout poacher *Brachyopsis rostratus*, a disulfide-bond-rich globular protein exhibiting high structural similarity with a carbohydrate-recognition domain of C-type lectin (Mw = 14 kDa), and AFP III from notched-fin eelpout *Zoarces elongatus Kner*, another globular protein composed of twisted loops folded into triple-strand β-sheets (Mw = 6.5 kDa)^15–17^. Fungal AFP was also identified from a mid-latitude snow-mold *Typhula ishikariensis*, whose 8^th^ isoform (Tis8) exhibited a strong ability of ice binding, and constructs a 7-ladder β-helical structure with a triangular cross-section (Mw = 22 kDa)^18,19^. In contrast, a defective isoform of AFPIII, whose A20 is replaced with Leu (A20L), was suggested to bind only weakly to ice crystal surface^20^. Figure 1 compares the three-dimensional structures and ice-binding sites determined for these five AFP molecules (AFPI-III, Tis8, and A20L). Such dissimilar molecular architectures should differentiate their ice-binding properties, which will help in the understanding of the key determinants of their IRI efficiency.

AFPs are commonly capable of binding to a single ice crystal composed water forming a hexagonal unit defined by the *a*_1_–*a*_3_ and *c*-axes, in which an ice crystal plane is represented by Miller–Bravais indices^1^. For example, the indices (0001), (10$\bar{1}$0), and ($20\bar{2}1$) represent a basal plane normal to the *c*-axis, a primary prism plane parallel to the *c*-axis, and a pyramidal plane defined by a sloped slice inclined by 14.9° to the *c*-axis, respectively. An AFP species targets specific ice crystal plane(s)^21^, which leads to the creation of a curved ice front on that plane between the bound AFPs, through the Gibbs–Thomson effect^22^. Such an ice front is energetically unfavorable for further adsorption of water, and its crystal growth is terminated^23^. This mechanism lowers the nonequilibrium freezing temperature (*T*_f_) and slightly elevates the equilibrium melting temperature (*T*_m_) of the AFP solution^24^. The resultant difference between *T*_f_ and *T*_m_ is termed thermal hysteresis (TH)^25^.

A technique named fluorescence-based ice plane affinity (FIPA) analysis is useful in determining the ice planes to which AFPs bind^26^. By submerging a single ice crystal hemisphere of 2–3-cm in diameter into a vessel containing a solution of fluorescence-labeled AFPs, their adsorptions show specific patterns on the ice hemisphere under UV light. If six equally distant ellipses are illuminated on the equator, they show that the AFP binds to six equivalent prism planes of a single ice crystal. Similarly, the illumination of six ellipses on the mid-latitude implies that the AFP is binding to six equivalent pyramidal planes. Entire illumination of the hemisphere implies that the AFP sample binds to multiple ice planes^26–28^. The present study examined the IRI efficiency of the five AFP samples, for which the time-dependent change of specific ice grains was analyzed to elucidate ice recrystallization kinetics during subzero temperature annealing. The relationship with their TH value and the FIPA pattern will be considered.

## Results and Discussion

**Figure 2:**
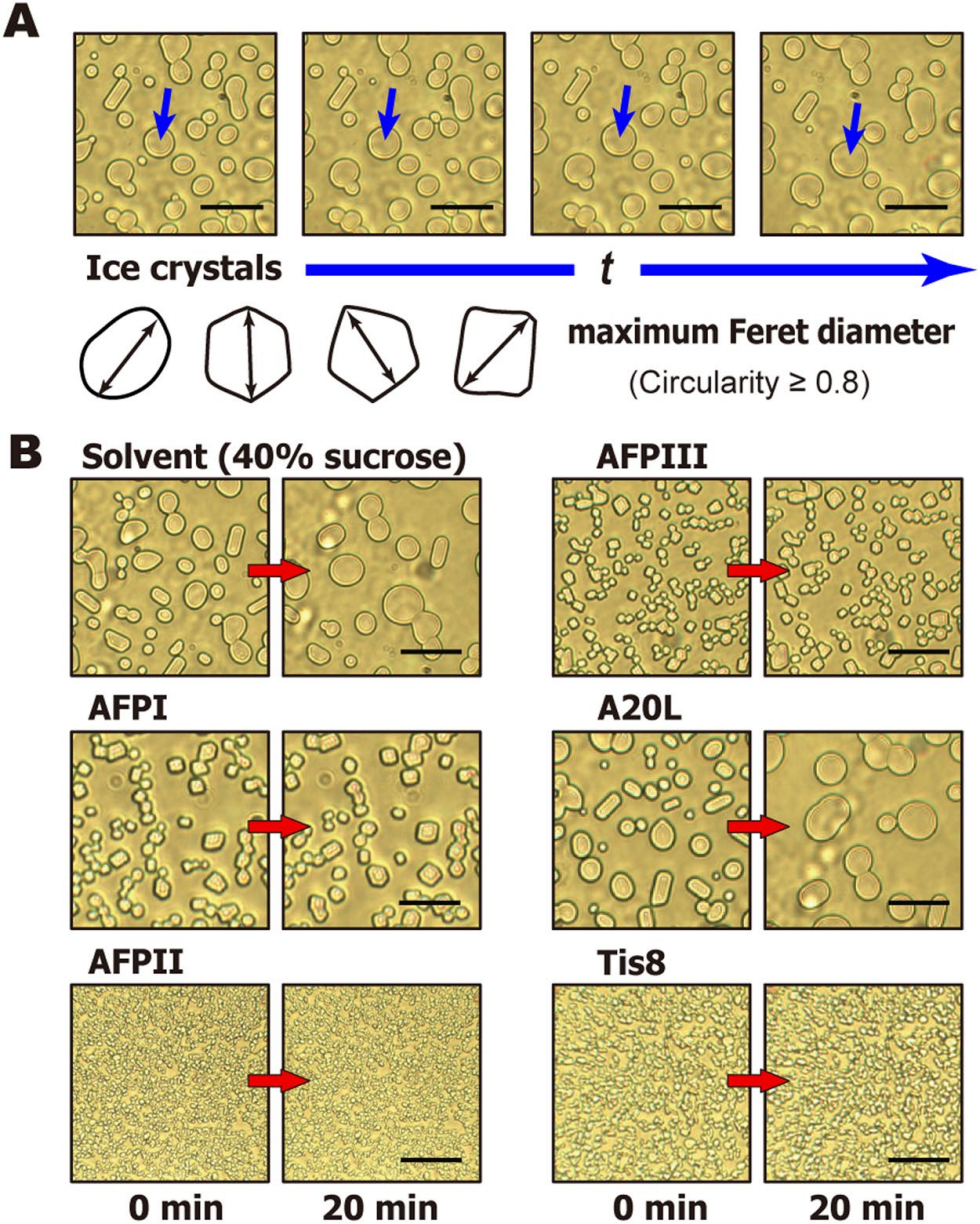
Photomicroscope snapshots of ice grains picked up from 40 min videos that recorded the ice recrystallization process. (**A**) Time-lapse image of an ice grain recorded in the last 20 min of the video file. It satisfies the criteria of 0.8–1.0 circularity and exhibited only uniform growth, from which the radius (*r*) was evaluated from their maximum Feret diameter (indicated with arrows) to be analyzed by the formal representation of Ostwald-ripening^32^. (**B**) Photo examples of time-dependent changes in the ice grains in solvent (40% sucrose) and 1.5 μM solutions of AFPI, AFPII, AFPIII, A20L, and Tis8 at −6 °C, taken from the last 20 min. The scale bars represent 50 μm.

**Figure 3:**
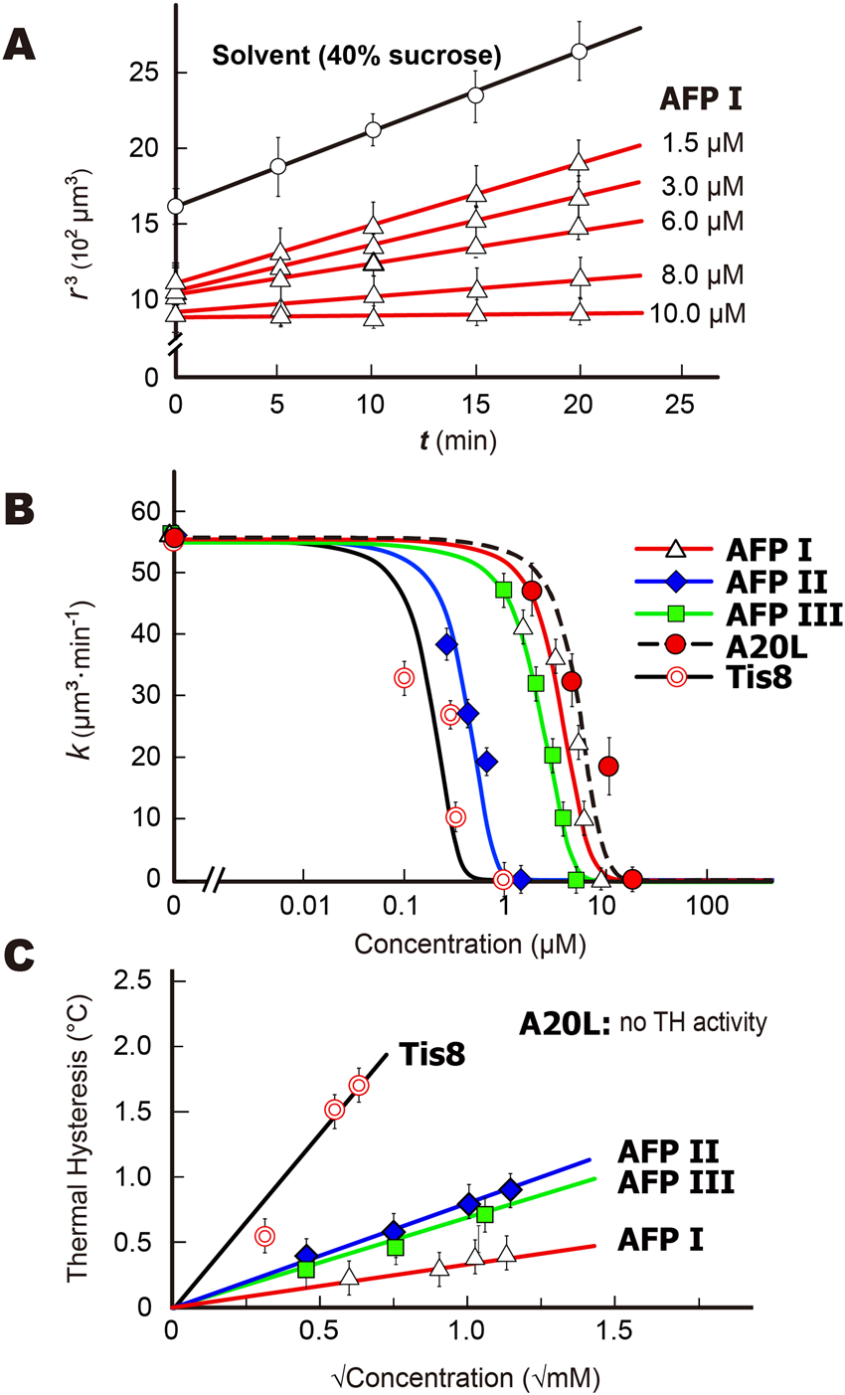
Determination of IRI efficiency for AFPI-III, A20L, and Tis8 samples. (**A**) Time-dependence of the radius cubed (*r*^3^) of 1.5, 3.0, 6.0, 8.0, and 10.0 μM solutions of AFPI, showing linear profiles. Such linearity was also detected for the solvent (40% sucrose). The slope shows the recrystallization rate *k* (μm^3^·min^−1^). (**B**) The S-shaped curves^33^ describing the concentration dependence of *k* value (μm^3^·min^−1^·M^−1^) for the five AFP samples. The inflection point (*C*_i_) is a parameter to compare the IRI efficiency between the samples, and the AFP concentration at k → 0 represents the IRI endpoint. (**C**) Concentration dependence of thermal hysteresis evaluated for the five AFP samples. The A20L failed to halt the ice crystal growth, for which no TH activity was evaluated.

The photomicroscope view of a sample was instantly darkened during the course of the initial sample cooling from room temperature to −40 °C, indicating that it was flash frozen to form a polycrystalline state composed of numerous single ice crystals. The ice crystals were tightly combined together at −40 °C, but melted to form a dispersion state on increasing the temperature to −6 °C. We captured a 40-min video of this dispersion state. Figure 2A shows an expanded view of the time-lapse images of the dispersed ice grains created in the solvent (40% sucrose without buffer detergents), whose sizes and shapes are not uniform. As shown, the ice grains underwent recrystallization with time (*t*) in different manners including; (i) uniform growth with keeping a relatively round shape (indicated by arrows), (ii) shrinkage by melting and disappearing, or (iii) merging to become larger ice grains (accretive recrystallization)^29,30^. The (i) and (ii) are known as migratory recrystallization^6^. These processes progressed consecutively in a variety of combinations to generate a range of diverse single and overlapping crystals. The time-lapse images of AFPI-III, A20L, and Tis8 whose concentration was adjusted to 1.5 μM, are also shown in Fig. 2B, where the ice crystals are more variable in shape and size depending on the AFP species. It is worth noting that if the average size of the ice grains is evaluated without any selection of the crystals, the value is highly biased by the choice of snapshot. Lifshitz and Slyozov^31^ originally deduced the Ostwald-ripening formula to describe the kinetic process, whereby diffusion effects cause the precipitation of grains of a second phase in a supersaturated solid solution^31^. We hence marked specific grains that increase in size by diffusion, while discarding the others that showed signs of the other two processes, (ii) disappearance and (iii) accretive recrystallization. A total of 13–15 ice grains were chosen from the last 20 min of the video files, whose ice volume fraction per all ice crystals are constant with time (*t*) (Supplementary Fig. S1). This suggests that these selected ice grains are statistical sample reflecting the whole system, although reshaping of ice crystals may have a contribution. The ice grains were not always round in shape but were modified into squares and polygonals (Fig. 2B). Most of the ice crystals in the reference solution (40% sucrose) exhibit the circularity above 0.8. We therefore set the circularity of ice grains in all solutions to be selected at 0.8–1.0. Image-J software (https://imagej.nih.gov/ij/) was employed for this analysis, which automatically extracts the area (A) and perimeter (P) of the ice grains to calculate their circularity (R = 4 πAP^−2^), where R = 1 implies perfect roundness. The radius (*r*) was calculated as the half-length of the longest distance of the ice grains, or the maximum Feret dimeter (Fig. 2A)^32^. A total of 13–15 ice grains were picked out from the video, whose radii were measured at 20, 25, 30, 35, and 40 min. The measurement was performed for 3–5 subsets of the 13–15 ice grains, and the mean value of all the subsets were plotted as time-dependent data at 0, 5, 10, 15, and 20 min (Fig. 3A). Videos were recorded for all AFP samples at various concentrations.

The known methods evaluated an averaged ice grain size from time-lapse snapshots from photomicroscope images, and determined the minimum AFP concentration necessary to halt ice recrystallization^11,33^. This was called the “IRI endpoint,” which has been used to evaluate the IRI efficiency of various materials besides AFPs, such as polysaccharides, small molecule carbohydrate derivatives, and non-antifreeze proteins^34–36^. Our video method also gives time-interval-snapshots of the ice grains to evaluate the IRI endpoint. The examples shown in Fig. 2B compare the grains observed for 1.5 μM solutions of the five AFP samples during a 20 min annealing period at −6 °C. As shown, larger ice grains are observed in the solvent (40% sucrose) as well as in the solutions of AFPI, AFPIII, and A20L, but not in AFPII and Tis8. The images of AFPI became like AFPII when we added more AFPI, for example, and the minimum concentration necessary to initiate such a visual change is evaluated at the IRI endpoint. Figure 3A shows an example of the time-dependence data of the *r*^3^ values (μm^3^) of ice grains examined in 1.5, 3.0, 6.0, 8.0, and 10.0 μM solutions of AFPI. The solvent data (40% sucrose) without AFP was also examined. The *r*^3^ values increased linearly with time (*t*), and their slopes represent the ice recrystallization rates (*k*) (10^2^ μm^3^·min^−1^), which became less steep with increasing AFP concentration (μM). A similar linear dependence was also obtained for the AFPII, AFPIII, Tis8, and A20L solutions. In addition to the IRI endpoint, ice recrystallization kinetics has been examined by fitting of a sigmoidal curve of the formula^33^ to a set of the *k* values determined at different AFP concentrations^11,33,37^. The semi-log plot version clarifies the lower concentration data, so that an inflection point in this plot has been used for determination of a unique concentration of AFP (*C*_i_) at which it is assumed to occur a turnover from diffusion-limited growth to liquid-to-ice-transfer-limited growth induced by ice-binding of AFP^33^. The *C*_i_ value hence represents the IRI efficiency of an AFP species. Here we observed that all of our data can be fitted by the S-shaped curves in the semi-log plot (Fig. 3B), implying that an increasing amount of the protein decreases the recrystallization rate to be terminated according to these curves. The *C*_i_ values are significantly different between the AFP samples, which are 0.27 μM (Tis8), 0.60 μM (AFPII), 3.00 μM (AFPIII), 4.69 μM (AFPI), and 7.69 μM (A20L), respectively. Olijve *et al*.^37^ evaluated the *C*_i_ values of 5.8 and 5.9 μM for recombinant proteins of AFPI and III, respectively, which are comparable to our data^37^. We also estimated 0.06 μM of the *C*_i_ value for native AFGP sample, for which we assumed an averaged molecular weight of 15 kDa although not clarified (Supplementary Fig. S2). The IRI endpoint is an approximate concentration when *k* becomes zero in the Fig. 3B curves, which is less than 1.5 μM for Tis8 and AFPII, while AFPIII, AFPI, and A20L require more large amounts. The approximate endpoints are 0.7, 11.0, 1.1, 8.6, and 12.0 μM for Tis8, AFP I–III, and A20L, respectively (Fig. 3B). These results support the information obtained in Fig. 2B. Yu *et al*.^38^, reported that the IRI efficiency is enhanced when AFPIII was dissolved in 0.1 M ammonium bicarbonate buffer (pH 7.9) or Tris-buffered saline (150 mM NaCl and 10 mM Tris-HCl) (pH 7.5), leading to a lower IRI endpoint at just 98 nM and 780 nM, respectively^38^. They also reported an extremely small value (0.16 μM) of the IRI endpoint for Sea raven AFPII with this the Tris-buffered saline, which also agree with the high IRI activity obtained for AFPII in the present study. Dissolved ions were assumed to recruit water molecules around themselves and reduce the amount of freezable waters that can join the ice crystal^38^, which may reduce the AFP consumption to halt the ice growth.

The TH value is the temperature range where an AFP-accumulated ice crystal neither grows nor melts. Technically, the TH evaluation needs to determine the difference between *T*_f_ and *T*_m_ of the solution, for which two types of techniques have been developed. One way is to observe a single ice crystal with a photomicroscope, where the stage temperature is computer-controlled to allow direct measurement of the growth-initiation temperature (*T*_f_) and the melting temperature (*T*_m_) of a single ice crystal. The other method is to detect the latent heat emission at the moments when the water freezes or melts, without observing the ice crystrals. Examples of the former method are to use a nanoliter osmometer or the previously reported photomicroscope system^39,40^, and the latter method includes differential scanning calorimetry and a sonocrystallization method^41,42^. In the latter method, heat emisson originates from an uncontrolled number of single ice crystals born in a solution at the moment of freezing. The number of surface-bound-AFPs per ice crystal will be hence be fewer, leading to smaller TH values compared with those from a photomicroscope-based method^37^. Here, we evaluated TH values of AFPI–III, A20L, and Tis8 from *T*_f_ and *T*_m_ determined by observation of a single ice crystal (Fig. 3C), where the crystal size (1.5 μm) and cooling rate (0.1 °C·min^−1^) were adjusted throughout the experiments. The highest TH value (2.2 °C) was obtained for Tis8, a hyperactive species of AFP^19^, which is 2–3 fold greater than that of AFPII (1.1 °C), AFPIII (0.9 °C) and AFPI (0.7 °C). These values are in good agreement with the TH values reported previously^23–25^. For A20L, its growth inhibition ability was not enough to arrest ice growth, no TH value was evaluated. Tis8 exhibited the strongest IRI (Fig. 3B), while its difference from the other AFPs was not consistent with the difference in TH. For example, the *C*_i_ values of AFPII and III (Fig. 3B) are significantly different, while their TH values are almost the same (Fig. 3C). Absence of correlation between TH and IRI was also reported in Olijve *et al*.^37^.

The mechanism of ice-binding has been thought to be based on the tertiary structure of the AFPs (Fig. 1). For example, AFPI is composed of three repeats of an 11-residue consensus sequence (Thr-X_10_, where X is mostly Ala) and constructs an α-helical structure^13^. On the basis of this property, AFPI protrudes four OH-groups on one side of this molecule at intervals of 16.5 Å, indicated by the yellow box in Fig. 1. This area includes consecutive Ala residues and is capable of binding complementarily to ice pyramidal planes where the oxygen atom spacing is 16.7 Å, and was assigned as an ice-binding site (IBS). Such IBSs are similarly constructed on each AFP structure, which are chartacterized by a planar hydrophobic surface that incorporates several polar groups^14^. Two IBSs bound to the ($10\bar{1}0$) prism and ($20\bar{2}1$) pyramidal planes are uniquely located side by side in AFPIII^20^. With using the informaton of putative ice-binding residues and structural coordinates, we evaluated approximate size of IBS (Å^2^) for each AFP sample (Supplementary Table S1). The size order was Tis8 (1544 Å^2^) > AFPII (1149 Å^2^) > AFPIII (820 Å^2^) > A20L (696 Å^2^) > AFPI (564 Å^2^)^16–20,43^. The ice-binding mechanism has recently been attributed to a semi-clathrate water network on the protein, but not the amino acid residues constructing the IBS; it merges with and freezes to a disordered water layer constructing the ice crystal surface^44–46^. Such water network may determine the ice-binding specificity of the present AFPs characterized based on the ice crystal morphology and the FIPA pattern.

**Figure 4:**
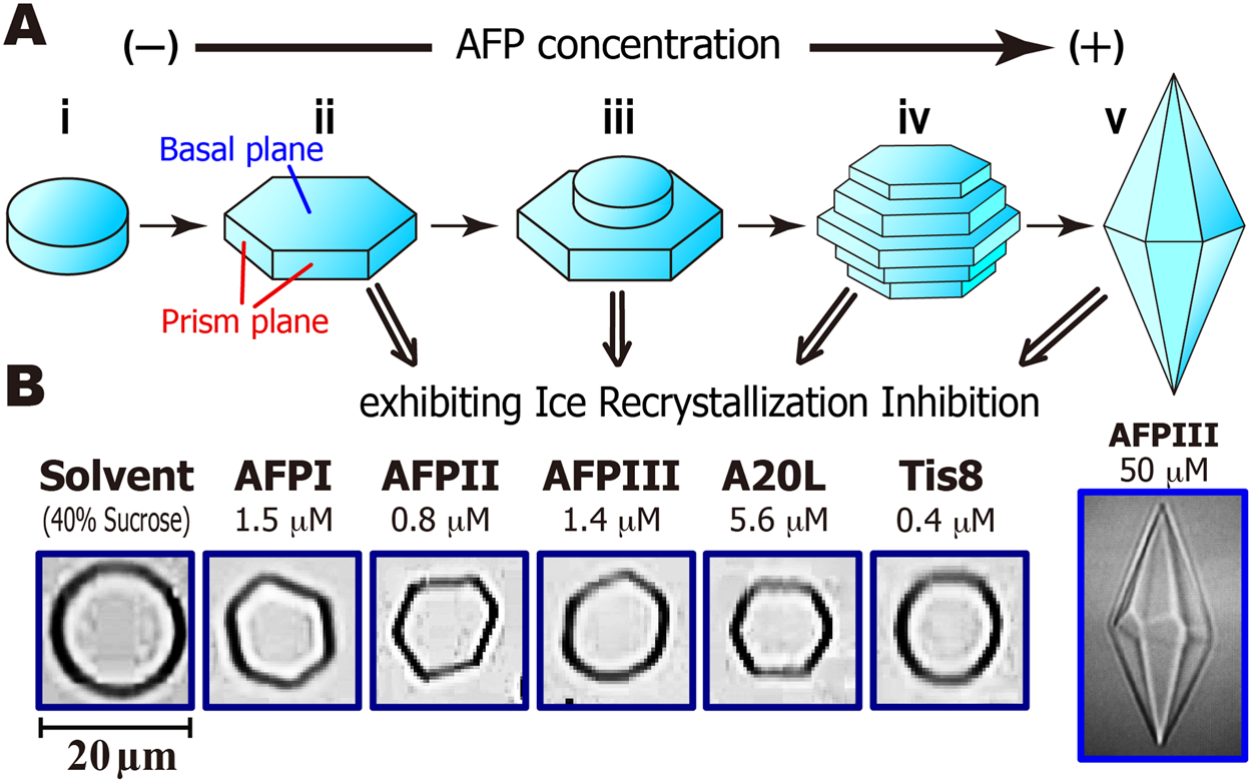
Relationship between the morphology of a single ice crystal and AFP concentration. (**A**) The crystal forms a round disk without AFP (i), while its six prism planes become visible by AFP-binding to form a hexagonal plate (ii). Further additions of AFP generate a new disk on the hexagonal plate (iii), leading to the formation of a barrel-like ice crystal composed of hexagonal ice layers (iv), and ultimately create a bipyramidal ice crystal (v). (**B**) Morphology of ice crystals observed for AFPI-III, A20L, and Tis8 at concentrations lower than their IRI endpoint. The hexagonal ice plate is created in the solutions of AFPI-AFPIII and A20L, while it is not facetted for Tis8 and similarly for the solvent (40% sucrose).

A schematic diagram of the single ice crystal morphology modified through the AFP-binding is illustrated in Fig. 4A^23,47^. A single ice crystal, which is composed of hexagonally arranged waters, is rounded to form an ice disk without AFP (Fig. 4A,i). Six prism planes, become visible by AFP-adsorptions to the waters that construct the planes, leading to the construction of the hexagonal plate (Fig. 4A,ii). Further binding of AFP causes a new disk to form on the hexagonal plate (Fig. 4A,iii) through the mechanism of two-dimensional ice nucleation theory^48^. This new disk is also modified into a hexagon, and repetitions of crystal growth and new disk generation progress (Fig. 4A,iv), leading to the construction of the bipyramidal ice crystal (Fig. 4A,v)^49^. Here we examined what morphology of ice crystal forms when the AFP exhibits IRI activity. Figure 4B compares the photomicroscope snapshots of an ice crystal created in each AFP solution at a concentration below its IRI endpoint (Fig. 3B). As shown, the hexagonal plate was formed with AFPI-III and A20L (Fig. 4B), while a disk shape was formed for Tis8, similar to that observed in the solvent (40% sucrose). Note that hyperactive AFP, such as Tis8, can bind to multiple ice planes to modify an ice crystal into a rounded lemon-shaped morphology^23^. Cheng *et al*.^19^ observed hexagonal plate-like-morphology for 9.0 µM solution of Tis8 during the TH measurement^19^, while the Tis8 concentration exhibiting the IRI activity (0.4 μM) is much lower than that concentration. These results suggest that (1) AFP can shape ice crystals into hexagonal plates even in the presence of 40% sucrose, and (2) IRI activity is aroused by extremely small amounts of AFP that can modify the shape of ice crystals from disks to hexgonoal plates (Fig. 4A,i–ii). The higher AFP concentration that creates bipyramidal ice crystals (Fig. 4A,v) is not necessary for IRI activity at all, which explains the previous indications that 0.5–100 μg·mL^−1^ (14 nM–1.4 μM) of AFPIII mixed with known cryoprotectants improves the viability of cells and tissues after frozen storage^50–52^. The excess amount of AFPs are generally harmful, which might be due to the sharpened edges of the ice crystals formed at higher AFP concentrations (Fig. 4A).

**Figure 5:**
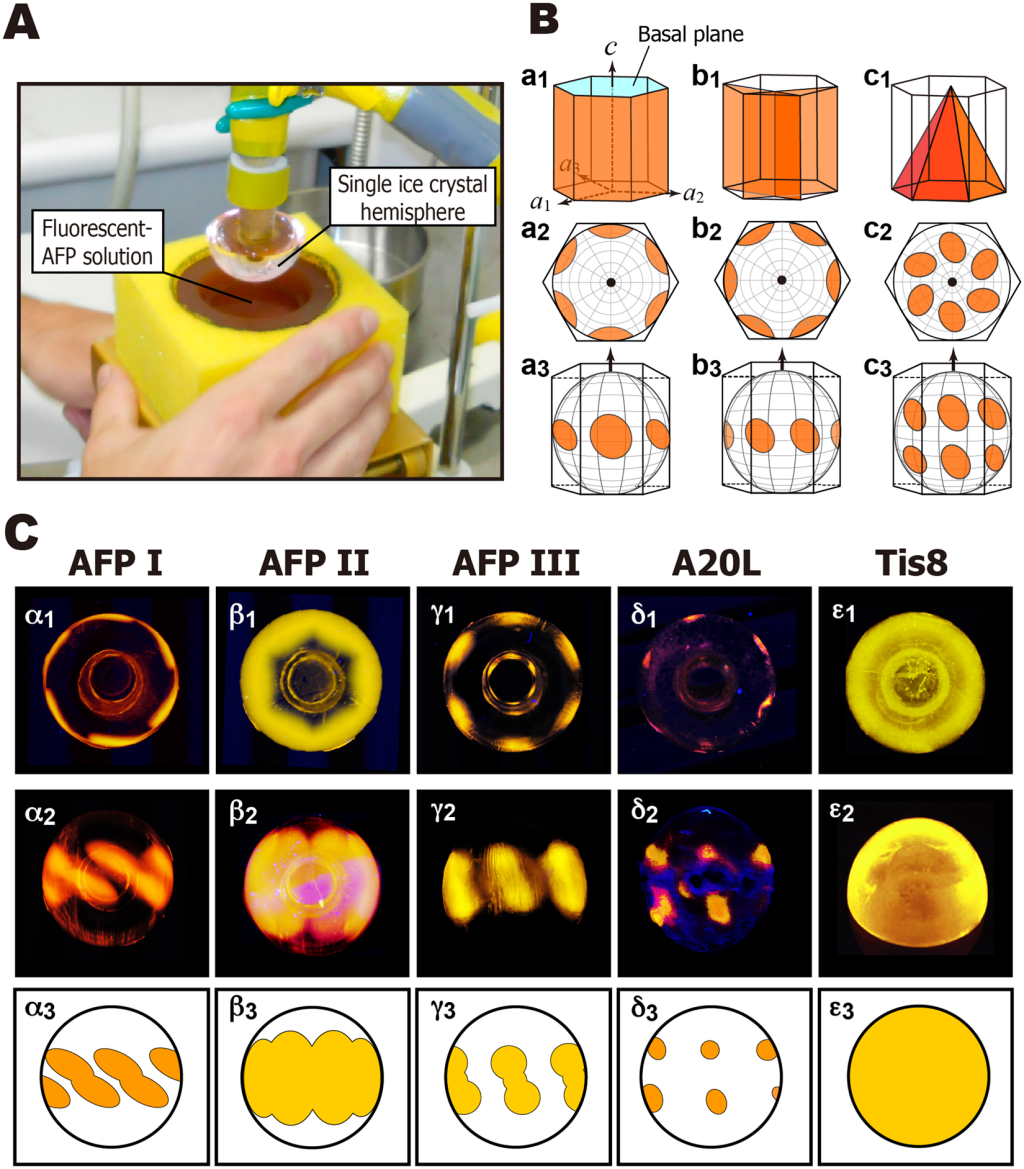
Analysis of fluorescence-based ice plane affinity (FIPA) of AFPI–III, A20L, and Tis8 samples. (**A**) Experimental setting to observe the FIPA pattern on a single ice crystal hemisphere. The ice is attached to a frosty probe, and immersed in a 0.1 mg·ml^−1^ solution of fluorescence-labeled AFP. (**B**) Relationship between the hexagonal ice unit and the FIPA patterns. The orange colors show the first prism planes (a_1_–a_3_), secondary prism planes (b_1_–b_3_), and pyramidal planes (c_1_–c_3_). The crystal graphic *c*- and *a*_1-_*a*_3_ axes are indicated by arrows in the illustrations, in which cyan in a_1_ indicates the basal plane. Illustrations a_2_–c_2_ are the patterns along the *c*-axis, and a_3_–c_3_ are those normal to the *c*-axis. (**C**) The FIPA images captured for the present AFP samples. The images α_1_–ε_1_ are the photographs along the *c*-axis, and α_2_–δ_2_ are those normal to the *c*-axis. The image ε_2_ shows an oblique view for Tis8. These images are translated to illustrations α_3_–ε_3_. The entirely illuminated FIPA pattern (ε_1_–ε_3_) was only observed for Tis8.

The FIPA pattern was observed on a single ice crystal hemisphere after soaking it in a 0.1 mg·ml^−1^ solution of a fluorescent AFP (Fig. 5A). Examples of the relationship between the hexagonal ice unit and the FIPA pattern is depicted in Fig. 5B, for which the ice hemisphere was attached to a frosty probe in two ways; one is to direct its *c*-axis downward (Fig. 5B,a_2_,b_2_,c_2_) and the other is to direct the 1^st^ prism plane downward (Fig. 5B,a_3_,b_3_,c_3_). When the 1^st^ prism planes (Fig. 5B,a_1_, orange) are targeted by AFP, the FIPA patterns in Fig. 5Ba_2_ (view along the *c*-axis) or a_3_ (view normal to the *c*-axis) are observed. For secondary prism planes (Fig. 5B,b_1_) and pyramidal planes (Fig. 5B,c_1_), patterns like b_2_ and b_3_, and those like c_2_ and c_3_, were observed, respectively. Their superposition creates a compound pattern for AFP that binds to multiple ice planes^20^. As shown in the left column of Fig. 5C, images α_1_ and α_2_ were photographed for the AFPI sample. The image α_2_ can be illustrated like α_3,_ which will help understanding of the relationship with Fig. 5B; the image α_2_ is created by a superposition of Fig. 5B-b_3_,c_3_. The Fig. 5C-α_1_ is similarly ascribed to Fig. 5B-b_2_,c_2_ that is close to the equator. As the consequence, the data suggest that AFPI binds to scondary prism planes (Fig. 5B,b_1_) as well as the pyramidal planes (Fig. 5B,c_1_) locating near the equator. AFPII exhibited FIPA in more wider area composed of multiple ice planes (Fig. 5C,β_1_–β_3_), while AFPIII binds to limited regions of the 1^st^ prism- and pyramidal planes (Fig. 5C,γ_1_–γ_3_). A20L binds to limited area in the pyramidal planes (Fig. 5C,c_1_–c_3_), which is the smallest among the present studied AFP samples. In contrast, the ice hemisphere is entirely illuminated with Tis8 (Fig. 5C,ε_1_–ε_3_), indicating that Tis8 binds to the whole ice crystal planes constructing the single ice crystal. When AFP samples are arranged in decreasing order of adsorption area, they from the sequence Tis8 > AFPII > AFPIII  ≥ AFPI > A20L. The same ranking was obtained when we compared their IRI efficiency (Fig. 3B), TH value (Fig. 3C), and approximate size of IBS, although a slightly larger IBS was estimated for A20L compared with AFPI (Table S1). Significantly, the weakest A20L exhibited no binding ability to the prism plane (Fig. 5C), but can shape an ice crystal into a hexagonal plate (Fig. 4B). All of the others, AFPI-III and Tis8, bind to the prism plane, while they can differentiate the IRI efficiency (Fig. 3B). These data suggest that the binding ability of AFP to a wider area of an ice crystal hemisphere determines the efficiency of its IRI, though their ice shaping abilities are indistinguishable from one another (Fig. 4B). The binding ability to higher-latitude pyramidal planes of AFPII, or the ability of Tis8 to bind to the basal planes, will terminate the diffusion-controlled absorption of water molecules to the hexagonal plate more entirely (Fig. 4A). This predicts that the IRI ability of AFPI, AFPIII, and A20L can be improved, if a constructive mutation is introduced into these AFPs to expand their adsorption area on the ice crystal.

## Materials and Methods

The samples of AFP I–III were purified from the barfin plaice, longsnout poacher, and notched-fin eelpout, respectively^15–17^. These fishes are living in the Okhotsk coastal area of Hokkaido island in the middle of winter, which were captured to prepare their muscle homogenates by Nichirei Corporation in 2016 (6-19-20 Tsukiji, Chuo-ku, Tokyo 104-8402, Japan). These homogenates were used as the source materials for our protein purification. The recombinant isoforms of fish AFPIII whose A20 is replaced with Leu (A20L) as well as a fungal AFP isoform from snow-mold *Typhura ishikariensis* (Tis8) were prepared using standard overlap extension PCR methodologies^17,19^. Briefly, *Escherichia coli* BL21 (DE3) containing each expression vector was cultured with Luria–Bertani medium, and the products were purified via cation-exchange chromatography (Econo-Pac High S column, Bio-Rad, USA) with a linear NaCl gradient (0–0.5 M) using 10 mM acidic buffer (pH 3.0). The five AFP samples (AFP I–III, A20L, and Tis8) were lyophilized after dialysis against Milli-Q water for overnight, whose purity was checked with SDS-PAGE and silver staining. Each sample was dissolved in degassed Milli-Q water containing 40% sucrose for the IRI measurements.

The ability of IRI was examined with a photomicroscope system described by Takamichi *et al*. ^40^. This system is composed of a Leica DMLB 100 photomicroscope (Leica Microsystems, Wetzlar, Germany) equipped with a cooling stage, whose temperature was manipulated between −196 °C and +25 °C with an accuracy of ±0.2 °C using a Linkam THMS600 temperature controller (Linkam Scientific Instruments, Surrey, UK). The ice crystal images were captured using a color-video 3CCD camera (Sony, Tokyo, Japan), and the temperature status was simultaneously viewed on a display to be saved as a video file. A 1 μl droplet of each AFP solution containing 40% (W/V) sucrose was sandwiched by two cover slips (ϕ = 13 mm) and set onto the stage. This sample was then frozen entirely by decreasing the temperature to −40 °C at a rate of −20 °C/min, and heating to −6 °C at a rate of 10 °C/min. Following exactly the same temperature manipulation, a video was captured to monitor the IRI process of the ice crystals at the temperature of −6 °C.

For evaluation of the TH value, the entirely frozen sample on the photomicroscope stage was heated until one single ice crystal was separately observed in the 1 μl AFP solution. The single ice crystal was then carefully cooled down or warmed up to observe its growth initiation or melting processes, whose critical temperatures were used as the nonequilibrium *T*_f_ and *T*_m_ values, respectively. A difference between the two temperatures was estimated as the TH value (i.e., TH = |T_m_−T_f_|)^40^.

The fluorescence-based ice plane affinity was also examined for the studied AFP samples according to the procedures described previously^20,26^. Briefly, a macroscopic single ice crystal of 2–3 cm diameter was initially prepared in a cylindrical mold. Following determination of its *c*-axis using a polarizer, a half-cut of this cylindrical crystal with a known orientation was mounted on a hollow copper tube (ϕ = 15 mm), in which −0.8 °C coolant was circulated by a refrigerant pump, Hitachi AMS-007 (Hitachi, Japan). A method called “ice pitting” generated a six-sided star mark on the polar region of the single ice crystal hemisphere, which indicates its *a*_1_–*a*_3_ directions. The hemisphere with known orientation is then mounted onto a chilled probe, so as to face the desired ice plane down. Following 1–2 hours incubation with a 0.1 mg/ml solution of a fluorescence-labeled AFP sample, the FIPA pattern illuminated on the ice crystal hemisphere was observed under UV light.

## Electronic supplementary material

Supplementary Information
