# Supplementary material for: Ice recrystallization is strongly inhibited when antifreeze proteins bind to multiple ice planes
Source: Sci Rep. 2019 Feb 13;9:2212. doi: 10.1038/s41598-018-36546-2 (PMC6374469; doi:10.1038/s41598-018-36546-2)
Supplement: Supplementary file 1 — Supplementary Information [file 41598_2018_36546_MOESM1_ESM.docx]

**Supplementary Information of**

Ice recrystallization is strongly inhibited when antifreeze proteins bind to multiple ice planes

Anika T. Rahman^1^, Tatsuya Arai^1^, Akari Yamauchi^1^, Ai Miura^2^, Hidemasa Kondo^1, 2^,

Yasushi Ohyama^2^, and Sakae Tsuda^1-3 *^

^1^ Graduate School of Life Science, Hokkaido University, Sapporo, 060-0810 Japan.

^2^ Bioproduction Research Institute, National Institute of Advanced Industrial Science and Technology (AIST), Sapporo, 062-8517 Japan.

^3^ OPERANDO Open Innovation Laboratory, National Institute of Advanced Industrial Science and Technology (AIST), Tsukuba, 305-8563 Japan.

Supplementary Figure S1


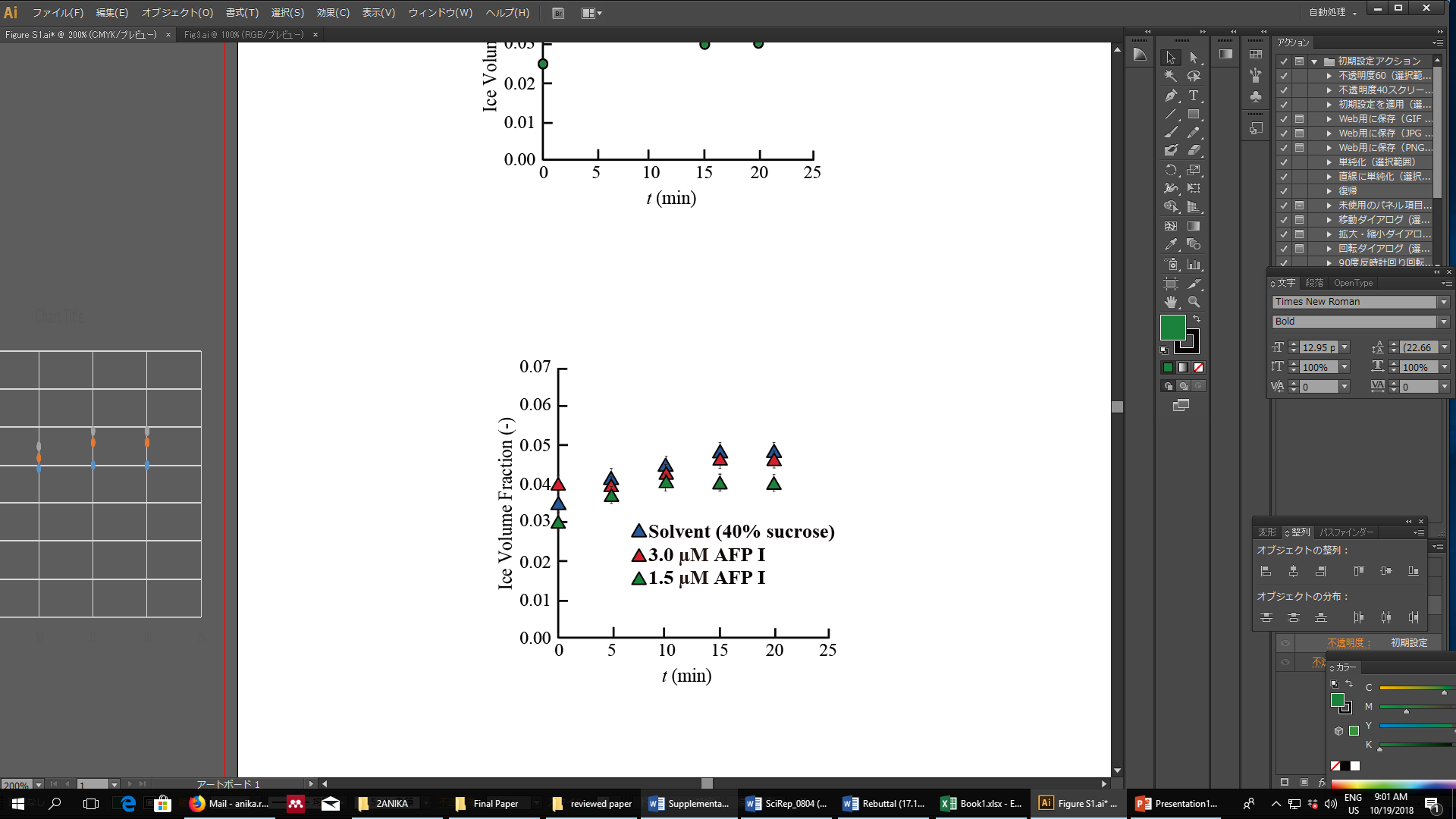


Figure S1: Time-dependence of the ice volume fraction during the IRI experiments. Ice volume fraction defined by ∑ area of 13-15 ice crystals / ∑ area of all ice crystals was evaluated for a reference solution (40w/w% sucrose, blue triangle) as well as the AFPI (BpAFP) solution at the concentrations of 3.0 (red) and 1.5 μM (green).

Supplementary Figure S2


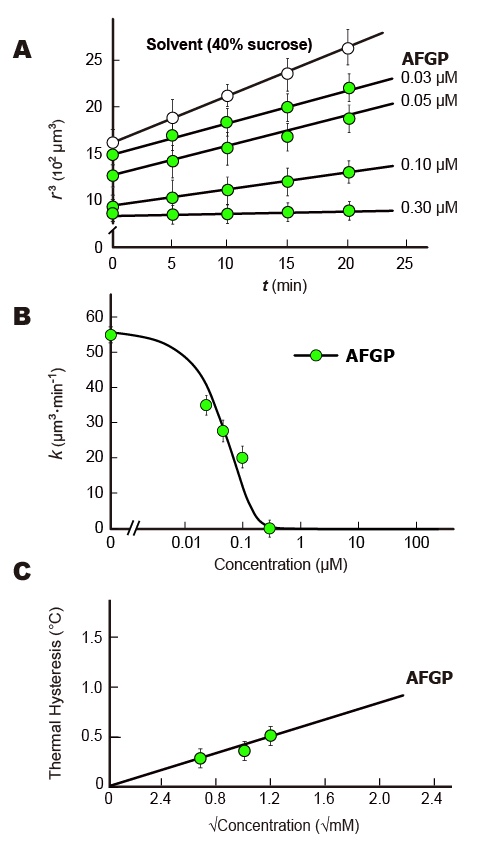


Figure S2. Preliminary IRI data of native AFGP sample (isoform mixture) purchased from NICHIREI Corporation, Japan. An averaged molecular weight of 15 kDa was assumed. A. Time-dependence of the cubed radius (*r*^3^) of 0.03, 0.05, 0.10, and 0.30 μM solutions of AFGP. B. Semi-log plot of *k* per molar concentration of the AFGP sample. The estimated *C*_i_ value and approximate IRI endpoint are 0.06 μM and 0.4 μM, respectively. C. Concentration dependence of thermal hysteresis (TH) determined for this AFGP.

Supplementary Table S1

| Residue of  AFPI | Area (Å^2^) | Residue of  AFPII | Area (Å^2^) | Residue of  AFPIII | Area (Å^2^) | Residue of  A20L | Area (Å^2^) | Residue of  Tis8 | Area (Å) ^2^ |
| --- | --- | --- | --- | --- | --- | --- | --- | --- | --- |
| Thr2 | 88 | Ile58 | 53 | Gln8 | 74 | Gln8 | 74 | Pro38 | 104 |
| Ala6 | 59 | Pro87 | 107 | Leu9 | 73 | Leu9 | 103 | Ala39 | 16 |
| Ala10 | 60 | Thr88 | 120 | Ile12 | 79 | Ile12 | 88 | Phe43 | 117 |
| Thr13 | 37 | Lys89 | 68 | Asn13 | 83 | Asn13 | 77 | Ser19 | 18 |
| Ala17 | 15 | Asn91 | 83 | Thr14 | 35 | Thr14 | 34 | Ala20 | 47 |
| Ala21 | 25 | Ile93 | 95 | Ala15 | 46 | Ala15 | 44 | Gly21 | 6 |
| Thr24 | 67 | Ser95 | 80 | Thr17 | 48 | Met20 | 23 | Ser23 | 40 |
| Ala28 | 36 | Asp96 | 48 | Pro18 | 48 | Val40 | 58 | Thr24 | 13 |
| Ala32 | 64 | Thr102 | 19 | Ala19 | 57 | Gly41 | 49 | Val25 | 67 |
| Thr35 | 113 | Ala103 | 74 | Met20 | 21 | Gln43 | 72 | Gln210 | 48 |
| Total | **564** | Ala104 | 40 | Val40 | 58 | Lys59 | 74 | Thr211 | 45 |
|  |  | Val105 | 122 | Gly41 | 54 | **Total** | **696** | Ala212 | 36 |
|  |  | Asp106 | 87 | Gln43 | 70 |  |  | Val213 | 2 |
|  |  | Leu112 | 44 | Lys59 | 74 |  |  | Ala214 | 32 |
|  |  | Ser117 | 72 | **Total** | **820** |  |  | Leu215 | 1 |
|  |  | His118 | 37 |  |  |  |  | Gln216 | 54 |
|  |  | **Total** | **1149** |  |  |  |  | Lys192 | 90 |
|  |  |  |  |  |  |  |  | Thr193 | 48 |
|  |  |  |  |  |  |  |  | Ala194 | 40 |
|  |  |  |  |  |  |  |  | Val195 | 3 |
|  |  |  |  |  |  |  |  | Thr196 | 40 |
|  |  |  |  |  |  |  |  | Lys198 | 75 |
|  |  |  |  |  |  |  |  | Ala174 | 36 |
|  |  |  |  |  |  |  |  | Gly175 | 14 |
|  |  |  |  |  |  |  |  | Ala176 | 44 |
|  |  |  |  |  |  |  |  | Val177 | 2 |
|  |  |  |  |  |  |  |  | Ser178 | 51 |
|  |  |  |  |  |  |  |  | Glu180 | 79 |
|  |  |  |  |  |  |  |  | Ala147 | 57 |
|  |  |  |  |  |  |  |  | Gly148 | 14 |
|  |  |  |  |  |  |  |  | Thr149 | 68 |
|  |  |  |  |  |  |  |  | Leu150 | 10 |
|  |  |  |  |  |  |  |  | Gly151 | 25 |
|  |  |  |  |  |  |  |  | Leu152 | 6 |
|  |  |  |  |  |  |  |  | Thr123 | 87 |
|  |  |  |  |  |  |  |  | Ser124 | 26 |
|  |  |  |  |  |  |  |  | Pro125 | 83 |
|  |  |  |  |  |  |  |  | **Total** | **1544** |

Table S1: Approximate size of putative IBS constructed in AFPI–III, A20L, and Tis8. The accessible surface area occupied by putative ice binding residues were listed and their summation was evaluated as the area of IBS for each AFP structure^16-20,43^. The AreaIMOL program of CCP4 software package^53^ was used for this evaluation.
